# Rice Galaxy: an open resource for plant science

**DOI:** 10.1093/gigascience/giz028

**Published:** 2019-05-18

**Authors:** Venice Juanillas, Alexis Dereeper, Nicolas Beaume, Gaetan Droc, Joshua Dizon, John Robert Mendoza, Jon Peter Perdon, Locedie Mansueto, Lindsay Triplett, Jillian Lang, Gabriel Zhou, Kunalan Ratharanjan, Beth Plale, Jason Haga, Jan E Leach, Manuel Ruiz, Michael Thomson, Nickolai Alexandrov, Pierre Larmande, Tobias Kretzschmar, Ramil P Mauleon

**Affiliations:** 1 International Rice Research Institute, DAPO Box 7777, Metro Manila 1301, Philippines; 2 Institut de recherche pour le développement (IRD), University of Montpellier, DIADE, IPME, Montpellier, France; 3 CIRAD, UMR AGAP, F-34398 Montpellier, France; 4 Advanced Science and Technology Institute, Department of Science and Technology, Quezon City, Philippines; 5 Department of Bioagricultural Sciences and Pest Management, Colorado State University, Fort Collins, CO 80523-1177, USA; 6 Indiana University, 107 S Indiana Ave, Bloomington, IN 47405, USA; 7 National Institute of Advanced Industrial Science and Technology, AIST Tsukuba Central 1,1-1-1 Umezono, Tsukuba, Ibaraki 305-8560, Japan; 8 Department of Soil and Crop Sciences, Texas A&M University, Houston, TX, USA; 9 Southern Cross Plant Science, Southern Cross University, Lismore, Australia

**Keywords:** rice, breeding, workflow, genomes, high-density genotypes, reproducibility, single-nucleotide polymorphism, genome-wide association studies, Galaxy project

## Abstract

**Background:**

Rice molecular genetics, breeding, genetic diversity, and allied research (such as rice-pathogen interaction) have adopted sequencing technologies and high-density genotyping platforms for genome variation analysis and gene discovery. Germplasm collections representing rice diversity, improved varieties, and elite breeding materials are accessible through rice gene banks for use in research and breeding, with many having genome sequences and high-density genotype data available. Combining phenotypic and genotypic information on these accessions enables genome-wide association analysis, which is driving quantitative trait loci discovery and molecular marker development. Comparative sequence analyses across quantitative trait loci regions facilitate the discovery of novel alleles. Analyses involving DNA sequences and large genotyping matrices for thousands of samples, however, pose a challenge to non−computer savvy rice researchers.

**Findings:**

The Rice Galaxy resource has shared datasets that include high-density genotypes from the 3,000 Rice Genomes project and sequences with corresponding annotations from 9 published rice genomes. The Rice Galaxy web server and deployment installer includes tools for designing single-nucleotide polymorphism assays, analyzing genome-wide association studies, population diversity, rice−bacterial pathogen diagnostics, and a suite of published genomic prediction methods. A prototype Rice Galaxy compliant to Open Access, Open Data, and Findable, Accessible, Interoperable, and Reproducible principles is also presented.

**Conclusions:**

Rice Galaxy is a freely available resource that empowers the plant research community to perform state-of-the-art analyses and utilize publicly available big datasets for both fundamental and applied science.

## Methods

We adopted the Galaxy framework to build the federated Rice Galaxy resource, with shared datasets, tools, and analysis workflows relevant to rice research.

### Background

With the decreasing cost of genome sequencing, rice molecular geneticists, breeders, and diversity researchers are increasingly adopting genotyping technologies as routine components in their workflows, generating large datasets of genotyping and genome sequence information. Concurrently international consortia have made re-sequencing or high-density genotyping data from representative diversity collections publicly available. These include but are not limited to the medium-depth (15–20× coverage) re-sequencing data of the 3,010 accessions from the 3K Rice Genome (3K RG) Project (∼1–2 million single-nucleotide polymorphisms [SNPs] per accession) [1, 2] and the 700,000 SNP Affymetrix array data for the 1,445 accessions of the High Density Rice Array (HDRA) germplasm collections [3]. The corresponding accessions are available at non-profit prices from the Genetic Resource Center of the International Rice Research Institute (IRRI) for phenotyping, allowing subsequent genome-wide association studies (GWAS) to be performed. Analysis of such datasets is a challenge to rice researchers owing to (i) the fairly large data matrix and the compute-intensive algorithms that require specialized computing infrastructure (a fairly large RAM, powerful central processing unit [CPU], and large disk space), and (ii) the relative difficulty in using open source/free software tools for analysis, which are commonly provided without graphical user interface and require proper installation in a Linux operating system environment.

On the computational side, public web resources with specialized tools already exist and are maintained at different institutions. The Rice SNP-Seek database [4, 5], largely developed and hosted by IRRI, contains phenotypic, genotypic, and passport information for >4,400 rice accessions from large-scale rice diversity projects such as the 3K RG and the HDRA collections. SNP-Seek [6] currently contains phenotype data for 70 different morphological and agronomic traits and stores SNPs and small insertions and deletions (indels) discovered by mapping the 3K RG accessions to 4 published rice draft genome assemblies, collectively resulting in the discovery of ∼11 million new SNPs and ∼0.5 million new indels. While SNP-Seek focused on delivery of prior analyzed content rather than providing an analysis platform, Gigwa [7, 8], hosted at the South Green portal [9, 10], is a scalable and user-friendly web-based tool that provides an easy and intuitive way to explore large amounts of genotyping data from next-generation sequencing (NGS) experiments. Gigwa allows for filtering of genomic and genotyping data from NGS analyses based not only on variant features, including functional annotations, but also on genotype patterns to explore the structure of genomes in an evolutionary context for a better understanding of the ecological adaptation of organisms. Gramene [11] is a curated, open source, integrated data resource for comparative functional genomics in crops and model plant species that, among other species, includes rice. Data and analysis tools are available as portals at the Gramene site [12]. In these resources mentioned, the analysis methodologies are custom-built by the respective projects.

There are other freely available web-based bioinformatics and breeding informatics software tools, optimized for plant species other than rice, including Araport [13] for *Arabidopsis*, Cassavabase [14] for cassava, and The Triticeae Toolbox (T3 [15]) for wheat and barley. While these tools are very useful, they are species/crop-specific and custom-built for the specialized requirements of their respective communities (such as project datasets), making adoption in rice challenging for ≥2 reasons: (i) the need to produce curated rice datasets that work seamlessly with the software system (e.g., genome-browser−ready data, curated genes, published quantitative trait loci from bi-parental crosses and GWAS and markers associated to traits), and (ii) the need for a dedicated software development team to customize the application for rice-specific data and analyses.

The ability of software to automate repetitive analysis task is attractive for data analysts, and the public sharing of the analytical methodology (as opposed to just the raw data and the results) enhances reproducibility and is being supported by academic communities of practice such as FORCE11 [16]. Many research groups working with NGS data have a high demand for computing infrastructure, and their complex analyses often comprise several steps using different software tools (pipeline). The deployment of these different software tools is a big challenge to small institutions without dedicated scientific computing support staff. There is no single solution to address these challenges. Our approach to help overcome them is the integration of a range of these different bioinformatics tools into the Galaxy bioinformatics system. Galaxy [17] is a web-based analysis workbench and workflow management system initiated at Penn State University. It includes a collection of software packages that can be operated via a web browser on a public server. Galaxy is a mature community effort, supported by various high-powered institutions, is relatively easy to deploy and maintain, and is thus well-suited to serve low- and moderately resourced institutions such as IRRI. The graphical user interface of Galaxy means that no knowledge of code is needed, thus facilitating bioinformatics analyses by researches without computational expertise.

**Figure 1:**
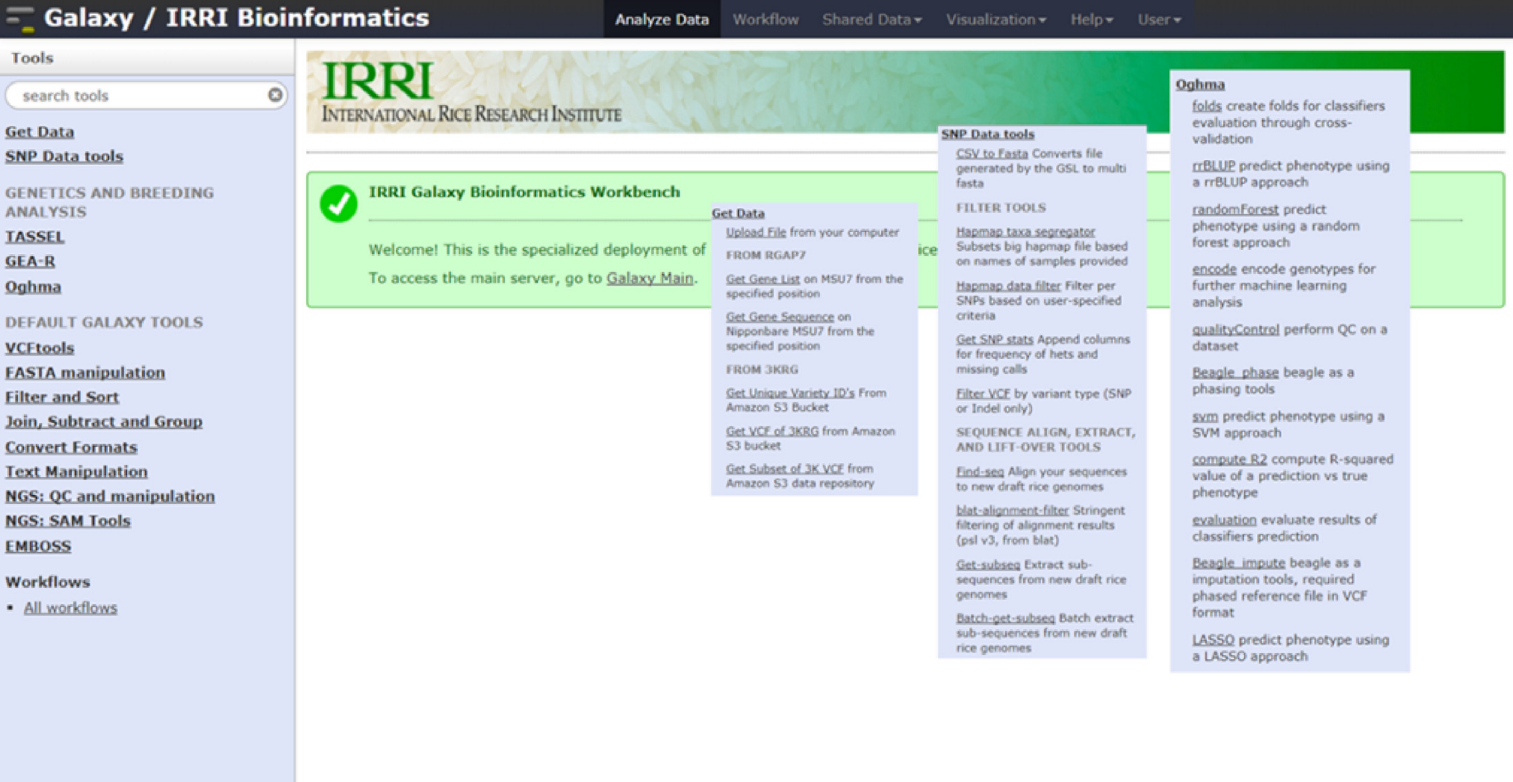
Rice Galaxy at IRRI with customized analysis tools for genetics, breeding, and custom data sources (i.e., 3000 Rice Genomes project).

We built a suite of federated Galaxy resources and tools, which we collectively named Rice Galaxy (Fig. 1). Rice Galaxy contains shared software tools and datasets tailored to the needs of rice researchers and breeders. A Rice Galaxy web server is also available, providing computing resources through an easy-to-use interface, and allowing reproducibility and publication of analytical methodology and results.

The Rice Galaxy federated resources are available at:

- Rice Galaxy reference web server, with working tools, built-in data, and shared datasets at IRRI [18];
- Rice Galaxy (common) Toolshed hosting the tool wrappers [19];
- Rice Galaxy code and built-in data for local/institutional deployment [20].

## Discussion

### Built-in/interoperable rice data

The Rice Galaxy system is customized to provide rice-specific genomic and genotypic data. Of primary importance is the gold-standard *japonica* variety reference genome (Nipponbare International Rice Genome Sequencing Project [IRGSP] release 1.0) [21], to which the reference gene models and most of the SNPs published have been anchored. In addition, 8 medium- to high-quality published genomes from various sequencing projects and the respective genome annotations for each are installed as alternative genome builds and are available as drop-down menu choices in Rice Galaxy. These include 4 high-quality builds from *indica*-type varieties Minghui 63 and Zhengshan 97 [22], IR 8 (GenBank: MPPV00000000.1), Shuhui 498 [23], as well as the *aus*-type variety N 22 (GenBank: LWDA00000000.1), as well as 4 medium- to low-quality genomes, 2 *indica* (IR 64 [24] and 93-11 [25]) and 2 *aus*-type rice genomes (DJ 123 [24] and Kasalath [26]). While these references were selected to represent diversity, they further represent variety groups that display agronomically important characteristics, such as heat and drought tolerance, disease resistance, submergence tolerance, adaptation to low-phosphorus soil, wide adaptability, good grain quality, aerobic (upland) adaptation, and deep roots [27–29]. Even though these genomes are highly similar to each other, they each contain unique regions (from 12.3 to 79.6 megabase pairs) that may harbor genes restricted to these variety-groups [5]. With the availability of several reference genomes, it becomes relatively straightforward to custom design SNP assays that are either of broad utility across varietal groups or specific to single groups.

Rice Galaxy includes genotyping data of the 3K RG (such as the 3K RG 3,024 accessions × 4.8 million filtered SNPs, 440,000 core SNPs, 1 million GWAS-ready SNPs, and 2.3 million indels) useful for GWAS, region-specific diversity analyses, and single-locus allele mining in the shared data library.

### Toolkits built (and detailed discussion of each toolkit)

#### SNP assay design: Lift-over of SNPs from one genome to another

SNPs discovered relative to the gold-standard reference genome (Nipponbare IRGSP 1.0 [21]) are commonly used in quantitative trait locus mapping (either by GWAS or biparental cross). In order to develop robust markers associated with the trait of interest, however, an SNP assay that works in the target varietal groups is needed. Consequently there is a need to “lift over” SNPs from one genome to another (e.g., from Nipponbare *japonica* to an *indica* varietal group represented by IR 64). The workflow is as follows: (i) get flanking sequences surrounding the target SNP in the source genome (the main reference Nipponbare); (ii) align these flanking sequences to the target genome of the variety of interest to verify whether it hits a unique region in the target genome of similar location from the source genome, allowing some mismatches but not allowing multiple region hits; and (iii) identify the flanking sequences surrounding the lifted-over SNP in the context of the target genome, for SNP assay design. The shared workflow is published in Rice Galaxy as "SNP liftOver," which runs smoothly in the public Rice Galaxy web server.

#### 3K RG data access

Rice Galaxy provides tools that can access the raw variant call format (VCF) files of each accession in the 3K RG project via connection (as data source in Rice Galaxy) to the 3,000 rice genomes at Amazon Web Services (AWS) Public Data [30], with tools allowing region-specific download. In Rice Galaxy, tools in the Get Data/FROM 3KRG section allow listing of the accessions in the 3K RG and retrieval of genotype data for a selected accession of interest from the 3K RG collection. The subset genome region of interest (chromosome name—base start—base end) can be specified and extracted from the VCF of the accession of interest stored in AWS Public Datasets. This functionality addresses a common use case for the 3K RG dataset, wherein a researcher has a gene or small genome region of interest mapped to the Nipponbare reference genome and wishes to determine the variation of this gene/genome region in a particular accession of interest from the 3K RG. As a result of the default limitations of the public Rice Galaxy server for user data storage space (∼6 GB), we recommend downloading subset regions instead of full VCF files (i.e., on average ∼2 GB) from the 3K RG collection. Analyses that require full VCF data of multiple 3K RG accessions using the Rice Galaxy server are not recommended; these are best performed using a local deployment of Rice Galaxy. The details of local deployment are discussed in the Rice Galaxy architecture discussion section.

In addition, we developed an original Rice Galaxy component called Rapid Allelic Variant extractor (RAVE), which allows simultaneous extraction of genotyping data from several accessions of the internal 3K RG resource. It relies on the PLINK software [31], which efficiently builds a user-adjusted genotyping submatrix from a compressed PLINK binary bi-allelic genotype table (bed file + bim, fam files). Users can customize the genotyping dataset vertically by choosing a subpopulation (e.g., *indica, japonica, aromatic, aus, tropical, temperate*) or setting a list of varieties, and horizontally by restricting variations with a list of genomic regions or a list of gene names. Additionally, users can filter the SNP positions by specifying thresholds for missing data or minor allele frequency. The extracted VCFs can be directly generated by Rice Galaxy, stored as output into the history pane of the Galaxy interface, and can be reformatted to Hapmap, a versatile file format for further analyses such as marker (SNP) design, GWAS analyses, or visualized in a JBrowse [32] genome browser (Vcf2jbrowse component). External SNP datasets can also be imported into Rice Galaxy and merged with 3K accessions in order to compare and look at the closest genotypes using the SNiPlay [33] workflow.

#### GWAS analysis using TASSEL

**Figure 2:**
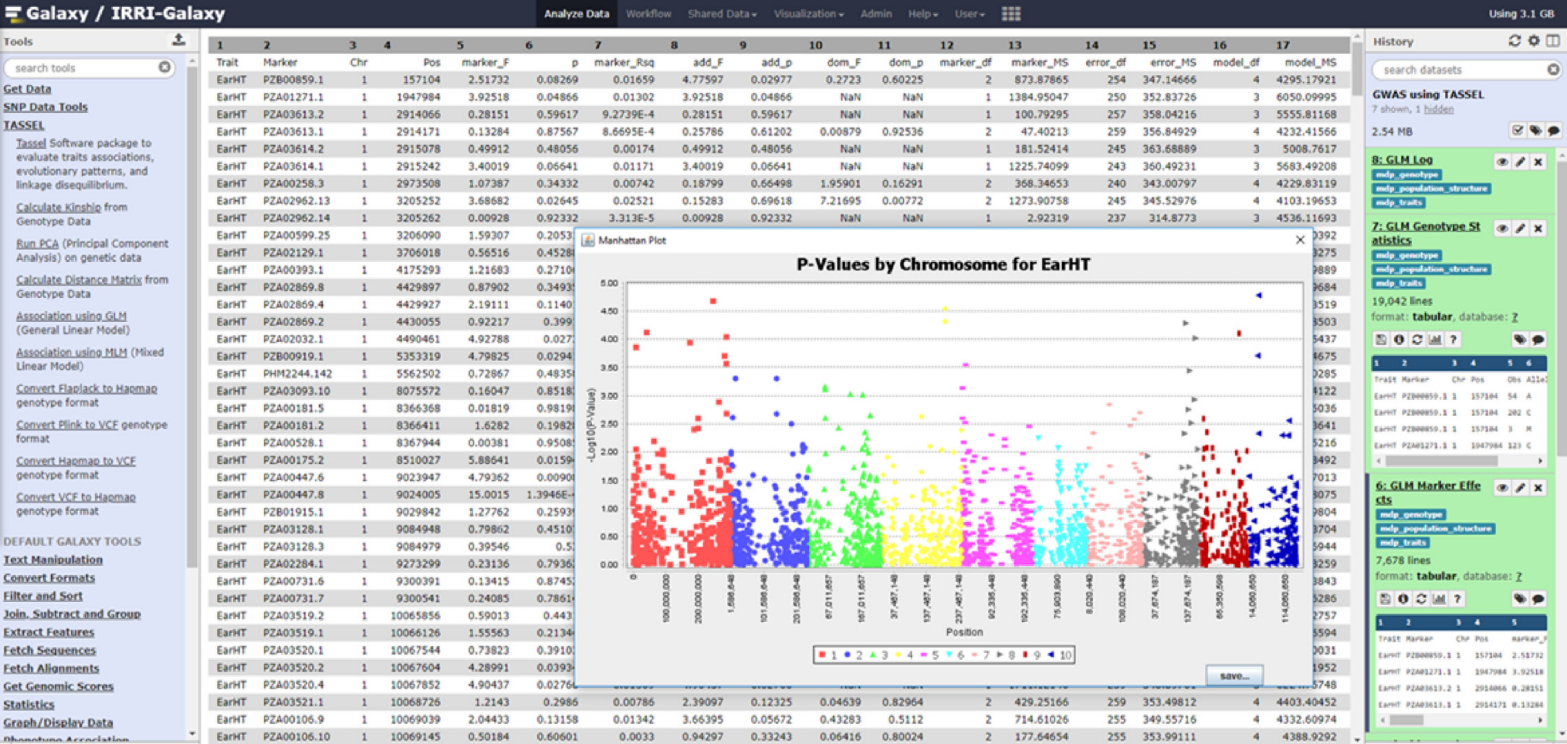
Genome-wide association studies analysis (implemented by TASSEL software) in Rice Galaxy.

**Figure 3:**
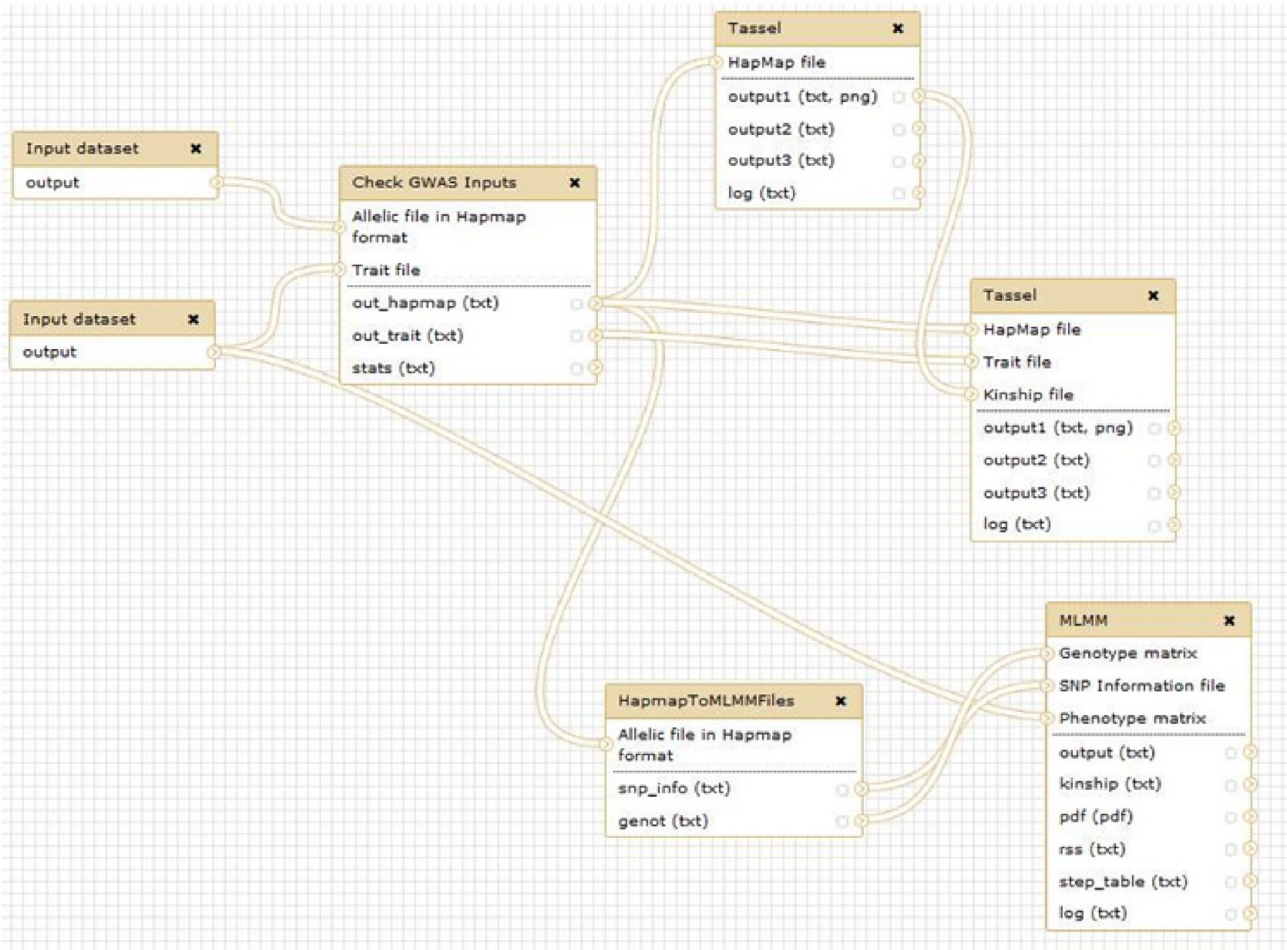
Genome-wide association studies analysis workflow in SNiPlay as implemented in Rice Galaxy.

The Rice Galaxy web server has sufficient storage and computing resources for GWAS, as long as the genotyping data are in matrix format (such as Hapmap), not as multi-sample VCF. Using this feature, it is relatively easy to construct a genotyping matrix for a subset of accessions from the 3K RG and connect associated phenotypic information to perform GWAS analyses online, with outputs being decorated with various graphical enhancements. For the 3K RG accessions, the subset 1 million GWAS and 440,000 Core SNPs that is usable for GWAS is already available as a shared dataset in Rice Galaxy (Fig. 2). Researchers working on the 3K RG panel can generate new phenotyping data from their respective experiments, upload the phenotype data into Rice Galaxy, and then perform GWAS using the TASSEL (Trait Analysis by Association, Evolution and Linkage) bioinformatics tool [34]. The GWAS Rice Galaxy workflow implementing TASSEL and Multi-Locus Mixed-Model package for association studies is shared from SNiPlay at Rice Galaxy (Fig. 3).

Aside from GWAS with 3K RG datasets, researcher-generated marker (emphasizing that it should be in matrix format) and phenotype data (outside of 3K RG) can also be uploaded to Rice Galaxy for GWAS analysis.

#### Genomic selection using Oghma genome prediction tool

**Figure 4:**
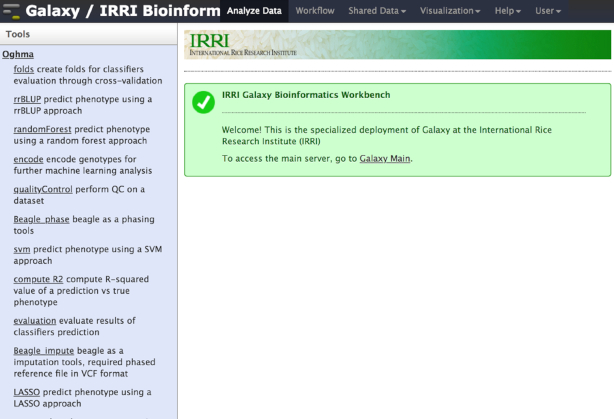
Oghma genomic prediction and selection tools in Rice Galaxy with various classifier tools installed.

The Rice Galaxy server allows the exploration of genomic selection methods. Genomic selection (GS) is a promising breeding technique with potential to improve the efficiency and speed of the breeding process in rice [35]. With the intent of enabling the GS analysis process used on the 2 datasets in the Spindel et al. [35] study (encoding data, filtering data to keep informative markers, creating a model from a training set, evaluating the model, and finally, performing the prediction itself), and to automate the analysis pipeline, the relevant packages (methods, fpc, cluster, vegan, pheatmap, pROC, randomForest, miscTools, pRF, e1076, rrBLUP, and glmnet) for the R Statistical language [36] were installed in Rice Galaxy and the tool suite was collectively named Oghma (Operators for Genome Deciphering by Machine Learning). A quality control tool (based on PLINK) and imputation tool using Beagle [37, 38] were also installed. Four phenotype prediction/classifier methods (ridge regression best linear unbiased predictor [rrBLUP], random forest, support vector machine [SVM], and lasso) were identified as relevant and deployed as tools in Rice Galaxy (Fig. 4).

**Figure 5:**
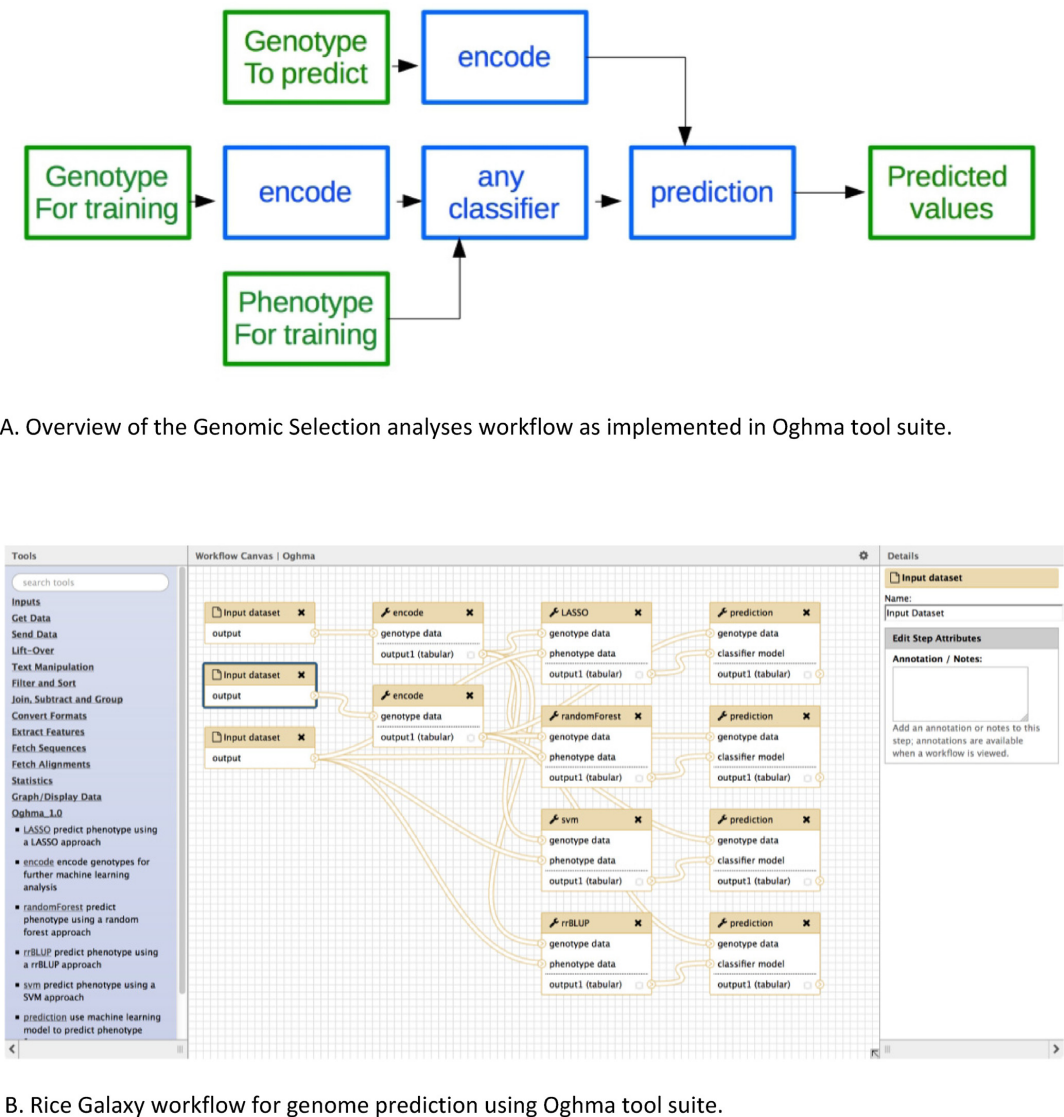
Genomic selection analysis workflow as implemented by Oghma tool suite.

Figure 5 shows the overall GS analysis workflow using Oghma. Genotypes are encoded through the "encode" tool. For the training set, an encoded genotype and the corresponding phenotype files are used by a classifier tool to train a model, which can be used with another encoded genotype file to predict trait values (the genomic prediction). It is important to note that (i) both the genotype for training and the genotype to predict must have the same markers (and thus, genotype files must have the same number of columns) to make a prediction, and (ii) the “evaluation” option of the classifier tool can have any value except 1 (it is recommended to keep the default value = 0).

A big challenge when using machine learning approaches for genomic prediction is the optimization of the model based on training data, specifically setting the best parameters of the aforementioned methods. Oghma was designed to automate the optimization of the parameter(s) of the classifiers on the fly (as opposed to manual tweaking), thus allowing users without experience of machine learning to easily optimize a model for their own data. Oghma includes some tools to evaluate prediction accuracy to allow the user to choose the most accurate method on their data by performing a cross-validation with a user-uploaded training set. Two metrics, the coefficient of determination (*R*^2^) and the correlation between predicted and observed phenotype, and a visualization (scatterplot of predicted vs observed) have been implemented to evaluate the methods. The "computeR2" and "plotPrediction" tools are used to compute *R*^2^ and visualize the accuracy of prediction. These tools both take the true phenotypes and the predicted outputs as inputs (note that both prediction and phenotype data must be in the same order) and return the computed *R*^2^ or the scatterplot display of true phenotype vs prediction.

**Figure 6:**
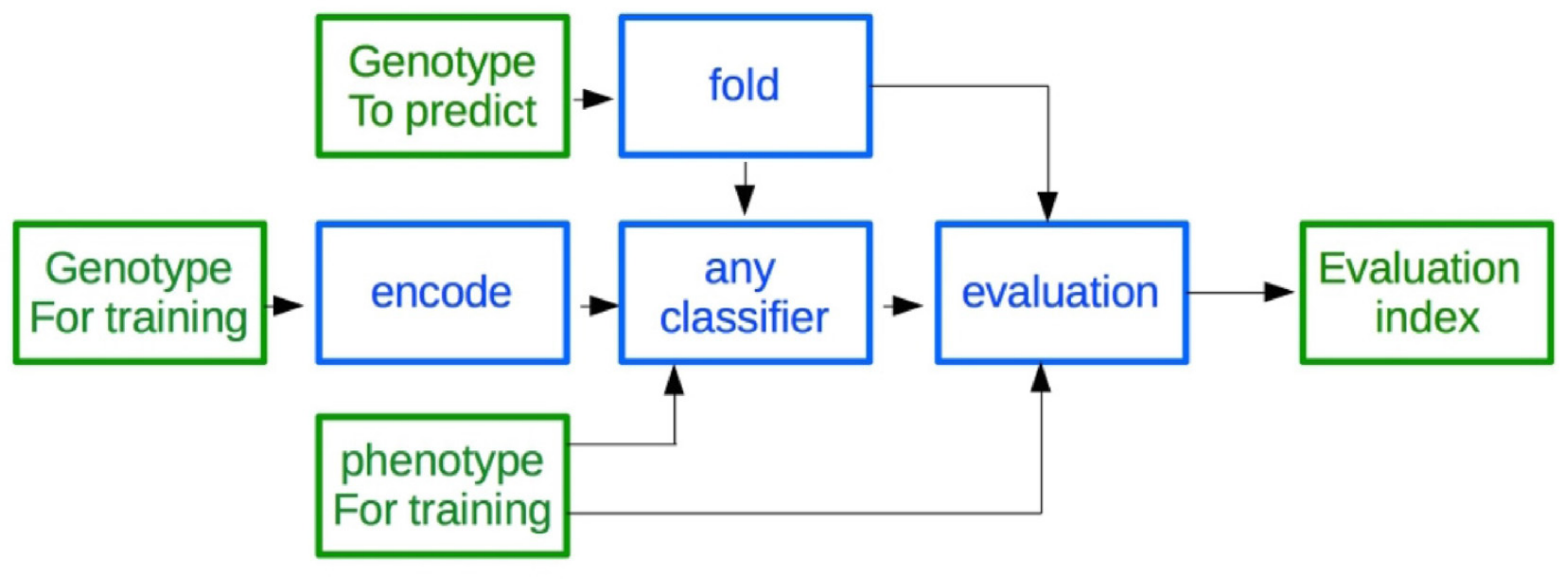
Workflow for classifier evaluation in the genome prediction tool suite implemented by Oghma.

Oghma can be used to evaluate a classifier (Fig. 6). Like the general GS workflow, genotype and phenotype are used as input for any classifier, but the “evaluation” option must be set to 1. Folds for cross-validations are designed through the "fold" tool, which takes as input the encoded file. These folds are used as extra argument by the classifier tools. The chosen classifier tool produces a file, which is not a model but the prediction of the test set for each cross-validation. This output is used as input, along with the phenotypes and folds, by the "evaluation" tool, which outputs some performance indices (*R*^2^ and correlation). Although it does give a real indication of performance, trying to predict the training set (i.e., using the same genotype file in the pipeline described above), or at the least, showing whether the classifier is not under-fitting the data.

We installed several classifiers in Oghma to allow users to test and determine the one(s) best suited for their dataset because our literature survey showed that no method seems to outperform the others on all genomic prediction tasks. It was noticed that random forest was the most accurate and the most stable classifier on the Spindel et al. dataset [35]; thus, we set this as the default in Oghma. An original aggregation method is also implemented in Oghma, aggregating outputs of multiple classifiers to improve prediction. This tool takes as input the prediction of *n* classifiers and tries to aggregate them through weighted mean of the prediction (weight optimized by genetic algorithm) or regression (multiple types of regression have been implemented, based on decision tree, SVM, and random forest). Limited testing has shown this approach to be promising, matching random forest in some cases, especially with a meta-SVM, with a polynomial or linear model as the aggregation method, but it still needs some improvement because the accuracy remains unstable when evaluated through cross-validation (data not shown). The aggregation method can also be evaluated using the aforementioned evaluation tools.

#### Diversity and population structure analysis of end-user datasets

**Figure 7:**
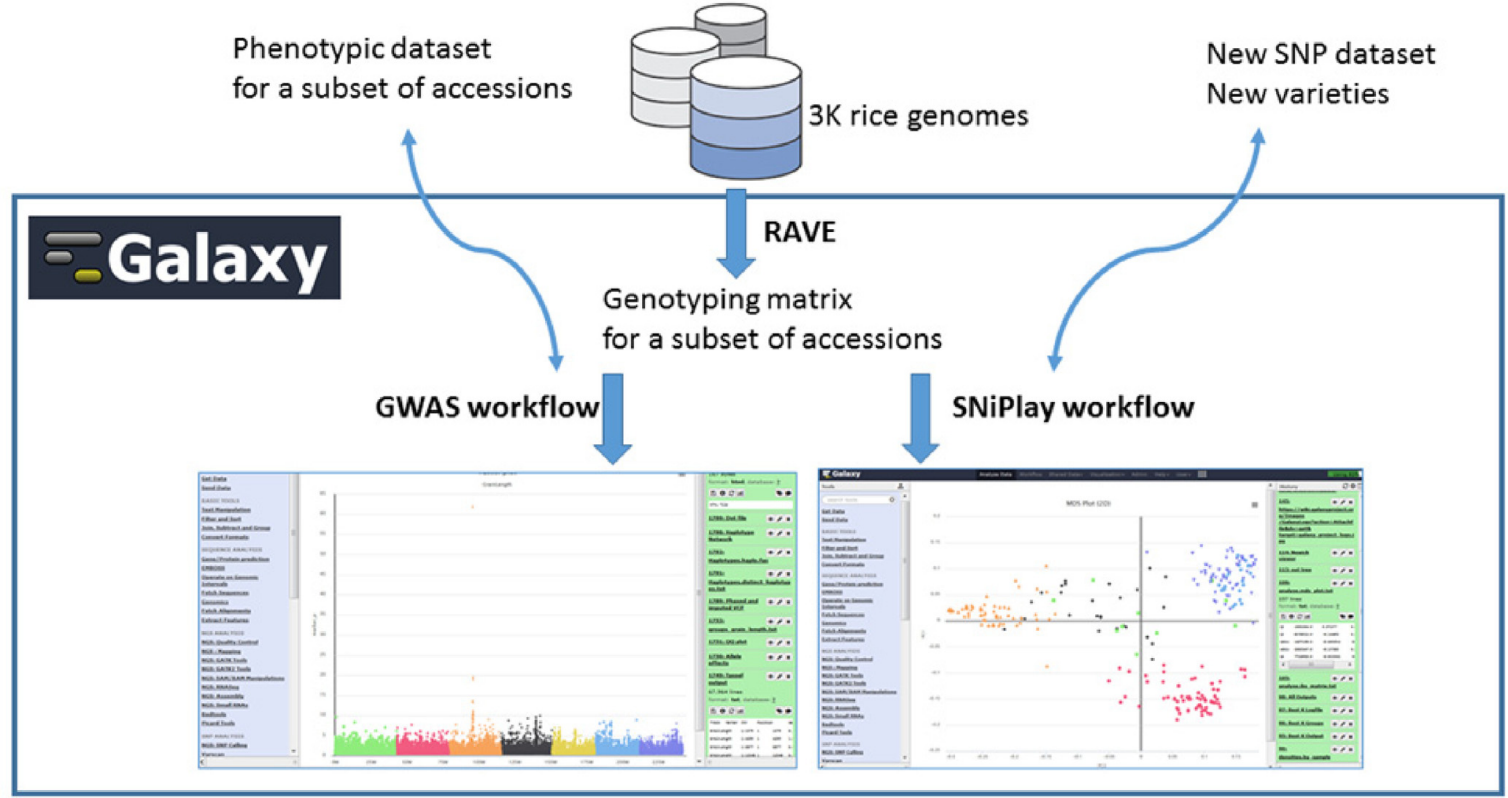
Overview schematic showing the integration of the 3K Rice Genomes project genotyping database and rapid extraction of subset SNPs by RAVE module for use by analysis workflows installed in Rice Galaxy.

**Figure 8:**
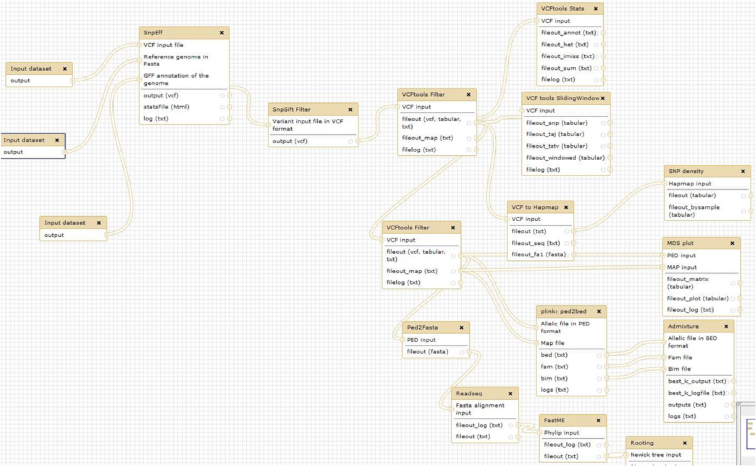
Rice Galaxy SNiPlay workflow for diversity and population structure analyses using various software tools.

Resources in the Rice Galaxy server allow diversity and population structure analyses. SNP datasets—such as those extracted from the 3K RG resource after filtering by the RAVE module or custom sets directly uploaded in the Rice Galaxy environment (Fig. 7)—can be processed for a complete exploration and large-scale analysis thanks to the SNiPlay Rice Galaxy workflow (Fig. 8). The workflow is available through the instance, requiring a VCF file as input. This workflow allows various analyses: (i) SNP annotation by snpEff [39] wrapper preconfigured for Rice Genome Annotation Project release 7.0 [40] gene models, (ii) variant filtration using the PLINK wrapper, (iii) general statistics such as transition-transversion ratio, levels of heterozygosity, and missing data for each variety using VCFtools, (iv) SNP density analysis, (v) diversity index calculation in sliding windows along the genome using VCFtools (Pi, Tajima's D, fixation index if subpopulations provided), (vi) linkage disequilibrium, (vii) population structure by sNMF [41], (viii) principal component analysis and identity by state clustering of varieties by PLINK, and (ix) SNP-based distance phylogenetic tree by FastME [42]. Most key steps are decorated with sophisticated visualizations using a dedicated plugin. Visualization can be displayed by clicking on the visualization icon.

In practice, this workflow can be processed for many applications such as the identification of possible introgression events, the identification of putative genomic regions involved in the control of qualitative traits through a fixation index approach, the investigation for potential duplicates in the 3K RG accessions dataset and custom datasets, or the estimate of closest varieties of new sequenced accessions, by ranking a list of varieties from the database most closely matching the given sample. It can also be used for the close inspection of a genomic region of interest after a GWAS analysis, through a linkage disequilibrium focus or the haplotyping of candidate genes.

#### Uniqprimer module

**Figure 9:**
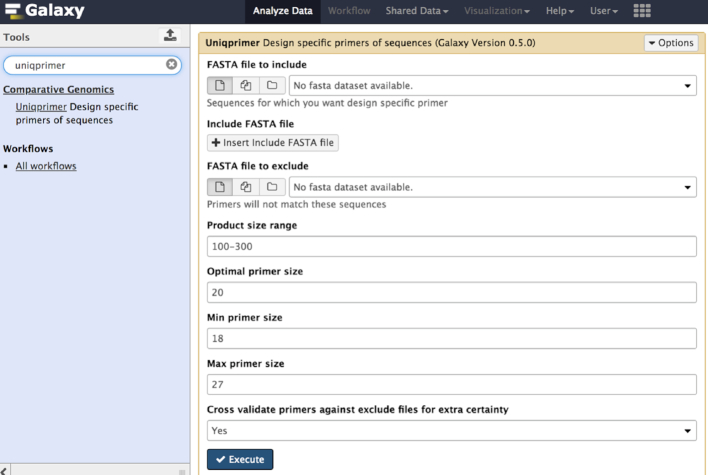
Uniqprimer comparative genomics−based diagnostic primer design tool for microbial pathogen detection installed in Rice Galaxy.

Uniqprimer is a workflow for comparative genomics−based diagnostic primer design, developed from a pipeline used in-house at Colorado State University to develop novel species- and subspecies-level diagnostic tools for bacterial plant pathogens including pathovars of *Xanthomonas translucens* [43], geographical variants of rice-associated *Xanthomonas* spp. [44–46], and the genetically diverse rice pathogen *Pseudomonas fuscovaginae* [47]. Uniqprimer is now deployed in Rice Galaxy for user-friendly diagnostic primer design from draft or complete pathogen genomes. The user inputs multiple bacterial genomes from diagnostic target species as well as non-target species (i.e., “include” and “exclude” genome files), and the tool performs comparative alignment, primer design, and primer validation to output a list of primers that are specific to the target genomes (Fig. 9). The Uniqprimer stand-alone program is written in Python and is available at the South Green github repository [48], along with detailed documentation for developers and end-users. The relatively small size of bacterial genomes allows the Rice Galaxy server to perform Uniqprimer analysis.

### Rice galaxy OA: a prototype for open access

IRRI, as a member center of the Consultative Group for International Agricultural Research (CGIAR [49]), complies with the CGIAR policy on Open Access and Open Data [50]. In collaboration with Indiana University in the United States and the National Institute of Advanced Industrial Science and Technology in Japan, and carried out through grants from the US National Science Foundation (NSF) and the MacArthur Foundation through the Research Data Alliance (RDA [51]), the team undertook a prototyping effort to bring the Rice Galaxy system to maximum compliance with the CGIAR policy.

The basis for the design to add open access (OA) to Rice Galaxy is a foundational technical idea emerging from activities occurring in the international RDA. This idea acknowledges that for open data access to be broadly realized, all meaningful data objects must have a globally unique and persistent identifier (PID). Globally unique means the name is not shared with other objects on a global scale. An identifier is persistent when the PID itself cannot be destroyed and when the relationship between the identifier and the data object it points to is permanent. Through an international working group in RDA, a team of researchers is advancing the notion of PID Kernel Information, which injects a tiny amount of carefully selected metadata into a PID record. This technique has the potential to stimulate an entirely new ecosystem of third-party services that can process the billions of expected PIDs. The key challenge of this working group is to determine which from amongst thousands of relevant metadata are suitable to embed in the PID record.

Our design draws on earlier work by us in data provenance capture and representation [52–54] and employs a hands-off technique ("data provenance capture") to gather information about a researcher's rice genomics analysis as the analysis is running. Through this technique, information acquired while the analysis is running is compiled and combined with pre-analysis information that is available at the beginning of the analysis workflow. Such information includes who performed the analysis, when it was performed, and under what conditions.

There have been earlier approaches to capture provenance of Galaxy workflows. Geocks et al. [55] developed a history panel for users to facilitate reproducibility. Gaignard et al. [56] propose the SHARP toolset, a semantic web (i.e., linked data) approach of harmonizing provenance collected from both the Galaxy and Taverna workflow systems. Kanwal et al. [57] captured the activity of a workflow (called a "provenance trace") including the version of analysis tools run, the software parameters used, and the data objects produced at each workflow step. This work also targets increased reproducibility of past workflow instances. Missier et al. [58] propose the “Golden Trail” architecture to describe and store workflow runtime provenance retrieved from Galaxy. The golden trail of provenance that is collected can be used to construct a virtual experiment view of past workflow runs. The 4 research contributions described further underline the need for the capture of provenance from workflow systems. They propose different but equally important uses of data provenance, i.e., to facilitate the improvement of science through reproducibility and construction of virtual views of an experiment once it has completed.

Our design for Rice Galaxy OA shares similarities with these other techniques; however, its end goal is different, which is to advance OA, hence making Rice Galaxy consistent with CGIAR's OA policy. To do this, we focus on each piece of data and information deemed valuable that emerges from workflow runs deemed to be of importance. These particular data and information must be retained and shared with others, while being subject to reasonable restrictions. This is a highly selective approach to provenance capture, and one that makes our work unique. We briefly outline the solution here and identify resources for those interested in pursuing the topic in more detail.

**Figure 10:**
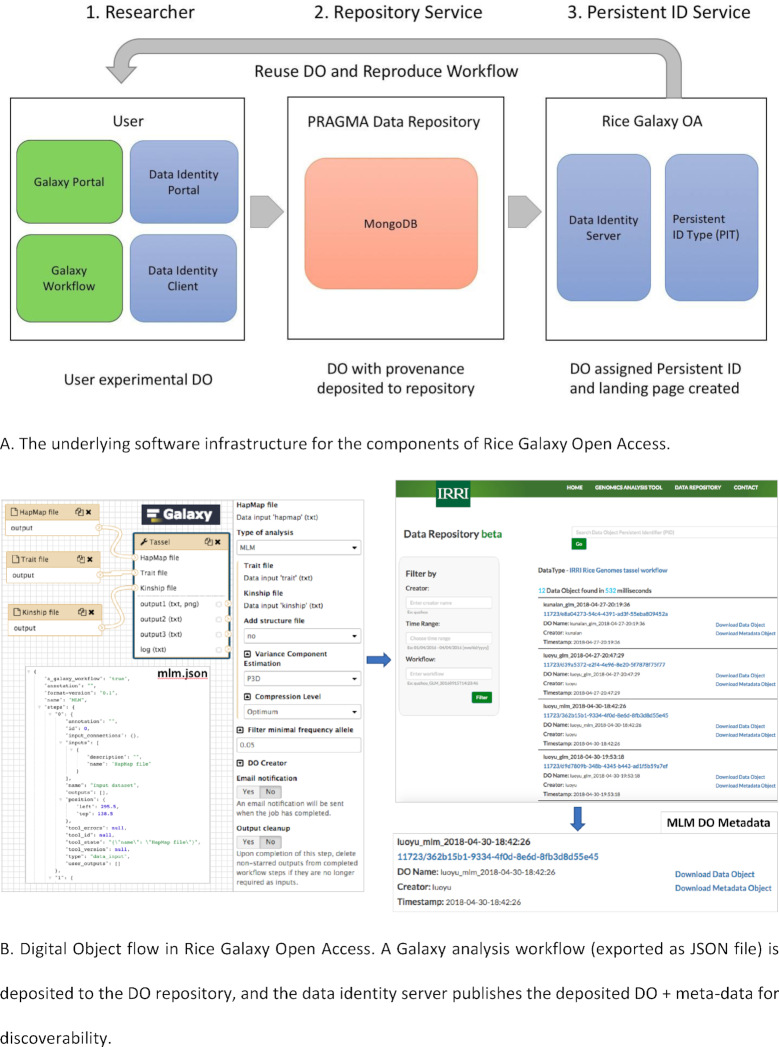
The components (**A**) and the flow of digital objects (DOs) from upload to discoverability (**B**) in the prototype Rice Galaxy OA.

The architecture of Rice Galaxy OA (Fig. 10A) utilizes the Handle system [59] and 2 standards emerging from the RDA, RDA PID Type [60] and the Data Type Registry [61]. It additionally uses storage and computing resources provisioned through the NSF-funded project Pacific Rim Applications and Grid Middleware Assembly (PRAGMA).

A researcher interacts with the OA-enhanced Rice Galaxy system as follows:

1. Researcher performs an analysis in Rice Galaxy.
2. Data objects (input data, output data, information such as configuration parameters) are extracted from Rice Galaxy OA into a PRAGMA Data Repository Database (MongoDB) (Fig. 10A).
3. The data objects are assigned PIDs, the PID Kernel Information is assigned into the PID record at this time, and a landing page is created for each (Fig. 10B).
4. Data objects can be downloaded from the Data Identity server and re-loaded to the Rice Galaxy server for full faithful reproduction of the analysis.

The resulting prototype system seems to be promising and addresses a number of the recommendations from CGIAR. The Rice Galaxy OA system is a user-transparent means of harvesting DOs from applications and assigning PIDs to scientific outcomes. The architecture is modular and built with default PID information types and metadata using RDA products (Fig. 10A). Although this proof-of-concept prototype successfully demonstrates the feasibility of this approach, there remains some future work. The community needs to provide feedback on which data and information products are most important to retain and make available. Additionally, not all workflow runs are important to a researcher becouse they could be system tests or new workflow tests. Thus, how a researcher identifies the items he or she wishes to make available to others, and when, remains an important consideration for this system. For more information, points of contact to the team, the underlying software for Rice Galaxy OA, and the link to the prototype server can be found at [62]. Note that Rice Galaxy OA is not implemented in the production Rice Galaxy server.

### Rice Galaxy architecture and deployment

We deployed the Rice Galaxy reference server (hosted by IRRI) into an AWS elastic computing cloud (EC2) instance (t2.large instance 2 vCPU, 4 GB RAM) with Linux Ubuntu release 12.04.2 LTS (GNU/Linux 3.2.0-40-virtual x86_64) operating system installed. We deployed Galaxy release 14 to this cloud server, following the method described in the Galaxy documentation. This Galaxy server has modest specifications and allows researchers to test the functionalities of the rice-specific tools installed, as well as conduct analyses on modestly sized datasets as allowed by the default memory and disk space allocation in the standard Galaxy deployment.

External data from the 3K RG Project files stored in the 3K RG AWS Simple Storage Service (S3) Public Data resource hosted at [63] (or s3://3kricegenome/) is accessed using AWS S3 Command Line Interface, a command line tool utility in AWS that provides an interface to access AWS S3 objects (CLI [64]). First, Rice Galaxy connects to the 3K RG AWS bucket using s3API and allows the objects inside the bucket to be transparent to Galaxy. VCF files (and the accompanying index files) are downloaded to Rice Galaxy using the S3 command line interface with the aws S3 cp command, executed as

aws –profile user s3 cp s3://3kricegenome/REFERENCE/VCF_FILE.snp.vcf.gz*.

The subset region of the VCF file (chromosome: start-end) is then extracted using BCFtools [65] wrapped in Rice Galaxy and exported to the history panel as a bgzipped, indexed BCF file, which can then be converted back to VCF using VCFTOOLS in Rice Galaxy.

**Figure 11:**
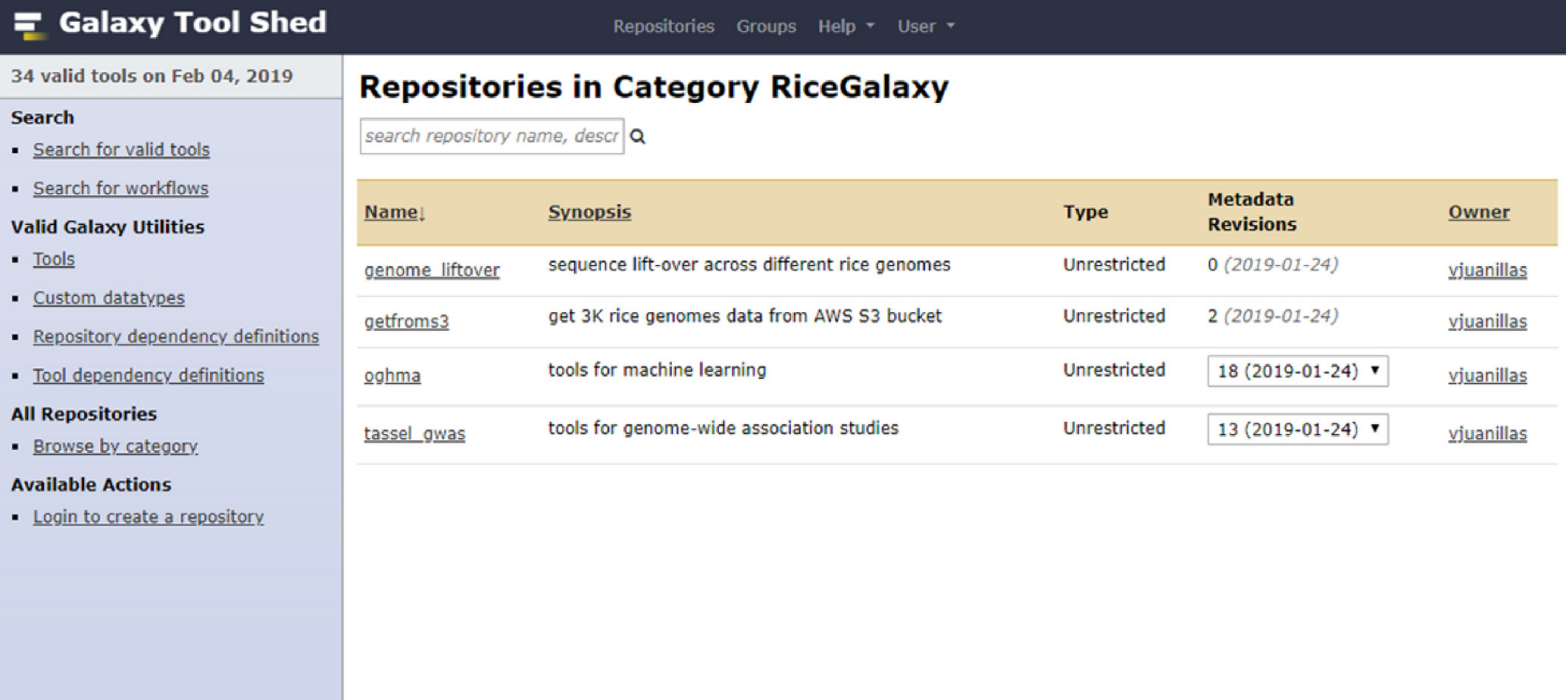
Rice Galaxy Toolshed with the various available tools.

Standard methods for tool wrapper development and deployment were followed. All tool wrapper XMLs developed specifically for Rice Galaxy are deposited and shared in a project-specific Rice Galaxy toolshed repository [19] (Fig. 11) and will also be deposited in the central Galaxy toolshed [66]. All developments and testing of Rice Galaxy and Rice Galaxy Toolshed were done in Docker containers hosted in virtual machines at the Advanced Science and Technology Institute, Department of Science and Technology of the Philippine Government (ASTI-DOST) prior to final deployment to the AWS instance.

In addition to the integration of these tools, new Galaxy wrappers and visualization plugins are being developed for visualizing chromosomes and their information (SNP density, structural variants, translocations) either in linear or circular mode, using recent web technologies (Ideogram.js [67], BioCircos.js [68], respectively).

We acknowledge that the reference Rice Galaxy server may not have sufficient storage and computing resources to allow analyses on multiple full-genome VCFs (e.g., full VCFs for 3 or more 3K RG accessions). We recommend the deployment of a local Rice Galaxy instance on a server that has more resources (RAM, disk space) and configuring Galaxy to provide access to the additional memory and disk space allocated to users. The general steps for local Rice Galaxy deployment are as follows:

1. Install Rice Galaxy and the required dependencies from the github repository mentioned in the Availability of source code and requirements section to your server.
2. Install the Rice Galaxy tools in your new Galaxy instance from the Rice Galaxy Toolshed; we are still developing these and will push the stable version(s) to the public Galaxy toolshed as soon as they are available.
3. Install the external tools from other projects that are installed in Rice Galaxy (but not in Rice Galaxy Toolshed, e.g., Uniqprimer, SNiPlay, RAVE, Oghma) to the local instance of Galaxy. Documentation on the availability and how to install these tools to local servers are available in their respective repositories and in the Rice Galaxy server Shared data →Pages section.
4. Download the shared 3K RG, test datasets, and tutorial pages from the Rice Galaxy server shared data library to your local Galaxy instance.

We are in the process of developing a Docker container of the Rice Galaxy server with tools following the Galaxy Docker flavor initiative [69] so that local server deployment is easier. The limitation of this method is that we cannot include third-party software in the container. The link to the container will be provided in the Rice Galaxy server once it is available. The institutions collaborating to build the Rice Galaxy system are committed to providing the installer, tools, data, and computing resources (however limited), in order to enhance or even drive the rice research community's respective institutional genetic/genomic/breeding efforts.

## Conclusion

Rice Galaxy is a federated Galaxy resource specialized for rice genetics, genomics, and breeding. The resource empowers the rice research community to utilize publicly available datasets (3K RG), materials (seed/accessions), and their own data, allowing complex data analyses to be performed even without investment in their own computational infrastructure and software development team. Rice research–related tools are also hosted on the Rice Galaxy server (i.e., Uniqprimer rice pathogen diagnostic design).

The Rice Galaxy system is freely accessible to all, and we invite the rice research community to participate in enriching the tools hosted by the resource. It can serve as a repository for data, analysis results, and new bioinformatics tools coming from institutions that have used the publicly available rice diversity panels from 3K RG, or have developed rice genomic/genetic analysis tools that they wish to share to the community, and a modest computing infrastructure for small institutes without in-house computing capability.

## Availability of source code and requirements

### Rice Galaxy

Project name: Rice Galaxy

Project home page: https://github.com/InternationalRiceResearchInstitute/RiceGalaxy

Operating system: Linux Ubuntu release 12.04.2 LTS

Programming language: Python

Other requirements: R release 3.2.3 and following packages: methods, fpc, cluster, vegan, pheatmap, pROC, randomForest, miscTools, pRF, e1076, rrBLUP, glmnet; TASSEL release 5.2.40; plink v1.90b3k; JBrowse 1.14.1; snpEff 4.3T; sNMF 1.2 (and as R package LEA); FastME 2.0

License: Rice Galaxy tools are released under GNU GPL. All software from external sources is bound by their respective licenses.

Any restrictions to use by non-academics: Rice Galaxy tools are without restriction to non-academics. All software from external sources is bound by their respective non-academic restrictions.

Code availability: Tool wrappers at Rice Galaxy Toolshed (http://galaxytoolshed.excellenceinbreeding.org). Rice Galaxy is available at IRRI Github (https://github.com/InternationalRiceResearchInstitute/RiceGalaxy).

### Uniqprimer

Project name: Uniqprimer

Project home page: https://github.com/SouthGreenPlatform/Uniqprimer

Operating system(s): Linux OS

Programming Language: Python

Other requirements: MUMmer 3

License: GNU GPL

Project name: PRAGMA Data Service

Project home page: repository https://github.com/Data-to-Insight-Center/RDA-PRAGMA-Data-Service/wiki/Welcome-to-PRAGMA-Data-Service-Prototype

Operating system(s): Platform independent

License: Apache License 2

## Availability of supporting data and materials

3000 Rice Genomes Project data are available from the *Gigascience* GigaDB repository [70]. Snapshots of the code and Docker images are also available from GigaDB [71].

3K RG BAM and VCF files are available from Amazon Public data and the ASTI-DOST IRODs site; see instructions at http://iric.irri.org/resources/3000-genomes-project.

SNP sets and morpho-agronomic characterization from 3K RG are available at the SNP-Seek download site (http://snp-seek.irri.org/_download.zul).

## Abbreviations

3K RG: 3000 Rice Genomes; ASTI-DOST: Advanced Science and Technology Institute, Department of Science and Technology of the Philippine Government; AWS: Amazon Web Services; CGIAR: Consultative Group for International Agricultural Research; CPU: central processing unit; DO: digital object; EC2: elastic computing cloud; GS: genomic selection; GWAS: Genome-Wide Association Studies; HDRA: High Density Rice Array; indels: insertions and deletions; IRGSP: International Rice Genome Sequencing Project; IRRI: International Rice Research Institute; NSF: National Science Foundation; NGS: next-generation sequencing; OA: open access; Oghma: Operators for Genome Deciphering by Machine Learning; PID: persistent identifier; PRAGMA: Pacific Rim Applications and Grid Middleware Assembly; RAM: random access memory; RAVE: Rapid Allelic Variant extractor; RDA: Research Data Alliance; rrBLUP: ridge regression best linear unbiased predictor; S3: Simple Storage Service; SNP: single-nucleotide polymorphism; SVM: support vector machine; TASSEL: Trait Analysis by Association, Evolution and Linkage; VCF: variant call format.

## Competing interests

The authors declare that they have no competing interests.

## Funding

Components of the project are supported by the following grants: Taiwan Council of Agriculture Grant to IRRI, International Rice Informatics Consortium, and CGIAR Excellence in Breeding Platform for financial support to the Rice Galaxy main server, the Bill and Melinda Gates Foundation through the Genomic Open-source Breeding Informatics Initiative project for applications development support, the USA National Science Foundation PRAGMA grant No. NSF OCI 1,234,983, the RDA/US-sponsored adoption program funded by the MacArthur Foundation, and the AIST ICT International Collaboration Grant.

## Authors’ contributions

V.J. and A.D. equally contributed to create Rice Galaxy. N.B. contributed the genomic prediction tools. A.D., G.D., P.L., and M.R. contributed the RAVE and SNiPLAY tools. J.D., J.R.M., and J.P.P. created the development Rice Galaxy cloud instances hosted at DOST-ASTI. L.M. created the SNP-Seek interfaces. L.T., J.L., and J.E.L. contributed the Uniqprimer tool. G.Z., K.R., B.P., and J.H. contributed the Rice Galaxy Open Access, M.T., N.A., and T.K. contributed to funding acquisition and writing, and R.P.M. coordinated the conceptualization of the project and the writing process.

## Supplementary Material

GIGA-D-18-00249_Original_Submission.pdf

GIGA-D-18-00249_Revision_1.pdf

GIGA-D-18-00249_Revision_2.pdf

Response_to_Reviewer_Comments_Original_Submission.pdf

Response_to_Reviewer_Comments_Revision_1.pdf

Reviewer_1_Report_Original_Submission -- Hiroaki Sakai 8/2/2018 Reviewed

Reviewer_1_Report_Revision_1 -- Hiroaki Sakai 9/12/2018 Reviewed

Reviewer_2_Report_Original_Submission -- Liya Wang 8/8/2018 Reviewed

Reviewer_2_Report_Revision_1 -- Liya Wang 9/19/2018 Reviewed

Reviewer_2_Report_Revision_2 -- Liya Wang 12/29/2018 Reviewed

## Editors Note

As one of the winners of the 2018 *GigaScience* ICG Prize, a video presentation is available from the journal website and youtube channel (https://youtu.be/HWKa3acoDUQ).
