# Corrigendum to: Rice Galaxy: an open resource for plant science

**DOI:** 10.1093/gigascience/giz156

**Published:** 2019-12-30

**Authors:** Venice Juanillas, Alexis Dereeper, Nicolas Beaume, Gaetan Droc, Joshua Dizon, John Robert Mendoza, Jon Peter Perdon, Locedie Mansueto, Lindsay Triplett, Jillian Lang, Gabriel Zhou, Kunalan Ratharanjan, Beth Plale, Jason Haga, Jan E Leach, Manuel Ruiz, Michael Thomson, Nickolai Alexandrov, Pierre Larmande, Tobias Kretzschmar, Ramil P Mauleon

In the original version of the article “Rice Galaxy: an open resource for plant science” by Venice Juanillas et al. [1], the main publication that generated the genome assemblies mentioned as important datasources in paper (IR 8: GenBank: MPPV00000000.1 and N 22: GenBank: LWDA00000000.1) was not included in the reference. In the online version of the paper, this occurs in the **Discussion/Built-in/interoperable rice data** section in the following sentence (highlighted in **BOLD** font):

“These include 4 high-quality builds from indica-type varieties Minghui 63 and Zhengshan 97 [22], **IR 8 (GenBank: MPPV00000000.1)**, Shuhui 498 [23], as well as the aus-type variety **N 22 (GenBank: LWDA00000000.1)**, as well as 4 medium- to low-quality genomes, 2 indica (IR 64 [24] and 93–11 [25]) and 2 aus-type rice genomes (DJ 123 [24] and Kasalath [26]).”

These two genomes should be cited using the reference indicated below[2] as reference 23, and we have apologized to the corresponding authors of this work and committed to them to make efforts to rectify this oversight. Following the sequence of references cited in the MS, it should now be written as (again highlighted in **BOLD** font:

“These include 4 high-quality builds from indica-type varieties Minghui 63 and Zhengshan 97 [22], **IR 8 [23]**, Shuhui 498 [24], as well as the aus-type variety **N 22 [23]**, as well as 4 medium- to low-quality genomes, 2 indica (IR 64 [25] and 93–11 [26]) and 2 aus-type rice genomes (DJ 123 [25] and Kasalath [27]).”

The authors sincerely apologize for these oversights.
