# Supplementary material for: Rice Galaxy: an open resource for plant science
Source: Gigascience. 2019 May 18;8(5):giz028. doi: 10.1093/gigascience/giz028 (PMC6527052; doi:10.1093/gigascience/giz028)
Supplement: Response_to_Reviewer_Comments_Original_Submission.pdf [file giz028_response_to_reviewer_comments_original_submission.pdf]

Author's Response To Reviewer Comments

Close

RESPONSE TO REVIEWERS REPORT:

Reviewer reports:

Reviewer #1 (HS): This manuscript describes the new open access data analysis platform, Rice Galaxy, based on Galaxy framework. It is of great importance to provide the service like this to make full use of the genotyping and phenotyping data derived from the 3k rice genomes project. I had no comments on the manuscript, but several issues related to the content of the Rice Galaxy. Please note that the following comments are based on the trial from the URL http://13.250.174.27:8080/, not http://galaxy.irri.org/.

Major issues
1. Since users are not necessarily familiar with the variety IDs such as 'B001' and 'IRIS_313-10000', variety names such as 'Heibiao' and 'SUWEON 311' should be acceptable. It would be very user-friendly if Rice Galaxy converts any variety names into corresponding unique IDs.

RESPONSE
Thank you for the very informative suggestion. As suggested, the RAVE tool has been improved to be more flexible so that it can accept both unique IDs (which we formally call assay ID) or variety names (aka Designation) in the list. Users are now allowed to provide a list of variety names in order to extract the corresponding SNPs.
The Get Data (FROM 3K RICE PROJECT) tool that accesses the AWS S3 bucket now allows using both assay ID and Designation, as suggested. The user just needs to select whether he/she is using ID or name in the selection button below the form prior to use (it is difficult to do auto-detection using AWS API so we programmed on the Galaxy server side).

2. It seems that only MSU gene IDs are valid to specify gene locus. If so, RAP gene IDs should also be acceptable.

RESPONSE
Thanks for this observation. We had to remove the Get Data (get gene list, get gene sequence) function in Rice Galaxy since this is duplicate of functions built in RAP DB (https://rapdb.dna.affrc.go.jp/tools/dump), and a gbrowse function in MSU7 (http://rice.plantbiology.msu.edu/cgi-bin/gbrowse/rice/), the original data source. We believe it’s best for the user to retrieve genes information from the authoritative sources, since they may periodically update the gene models / annotations, hence this data should be downloaded from the main web sites of these projects. As we implemented it, we copy the gene list and sequences from the two sources and store them internally in Rice Galaxy, hence this is bad practice since we have to update the information occasionally from the main source, and we also “hijack” queries that should be directly made to the authoritative sources.

3. Is there any reason for why only SOLiD data is subject to QC and manipulation tools? If Rice Galaxy outputs fastq files and/or enables users to analyze fastq files, additional tools such as QC (e.g. FASTQC), mapping to reference, and variant calling should be implemented. Otherwise, it would be better to omit the 'NGS: QC and manipulation' tools.

RESPONSE
Thank you for pointing this out, this is an error when we migrated from the original server at galaxy.irri.org to the newer one. Eventually we will deploy the newer server at the same URL. We have fixed the problem by removing all NGS QC related tools, as Rice Galaxy is not designed to analyze NGS

raw reads data, based on the resource allocation that we used for the server.

Minor issues
1. When users select 'Yes' for 'Filter SNP based on subpopulation' item in the 'RAVE' tool, more than one subpopulations should be able to be selected.

RESPONSE
Thanks for the suggestion. The RAVE tool has been improved accordingly to be able to select and combine more than one subpopulations.

2. The usage of the tool 'FROM 3K RICE PROJECT - Get Subset of 3K' tells to use 'convert formats-convert BCF to VCF tool' for further analyses, but such tool is not available in the toolbox. Is 'BCFtools-bcftools view VCF/BCF' the one?

RESPONSE
Thanks for pointing this out, you are correct, and we have made the necessary changes in the text of the info section of the tool.

3. The tool 'Oghma' was not found in the toolbox. Is 'Genomic Prediction' the one?

RESPONSE
Once again, thanks again for pointing this out, you are correct, and we have made the necessary changes in the text of the Genomic Prediction tool. It (Oghma) is now searchable.

Reviewer #2 (LW): This paper extends the SNiPlay workflows and provides a AWS powered Galaxy platform for the Rice community to leverage the Rice variation data. This could be useful but there are several significant concerns regarding the design of the system.

1. One challenge listed in the paper is that large CPU and RAMs are needed for dealing with fairly large data matrix. However, the entire Rice Galaxy platform is powered by an AWS machine that has only 2 CPUs and 4G RAM. Is this enough for large scale computation or the system is just built for small scale computation?

2. A related question is how long will this system last. Whats the cost of the system? If user wants to download all Rice VCFs from AWS using the Rice Galaxy, what's the cost of the data transfer? Its mentioned that S3 CLI is used to copy the entire gzipped VCF file to Rice Galaxy and then get subsetted with BCFtools. Is the copy free? It will be good to know how this is handled, otherwise, the system won't last long for serving the Rice community.

RESPONSE
Thank you very much for these very relevant questions. This is the combined response for these are as follows:

On the 1st sub-question, how long will Rice Galaxy last? IRRI is committed to keep the running Rice Galaxy server in AWS as long as funds from several projects that support it is available. It is currently funded by several projects: Two Bill & Melinda Gates-funded projects, namely, Genomics Open-source Breeding Informatics Initiative (http://gobiiproject.org/) which ends in 2019, concurrently funded by Excellence in Breeding (http://excellenceinbreeding.org/), which will be the overall program that absorbs GOBII after 2019 (it’s a 5 year program, starting in 2017). Another funding source is the Taiwan Council of Agriculture grant to IRRI, that has been stable over the last 3 years and is committed to continue in a 3rd phase starting 2020. The collaboration with the Computing and Research Environment service of the Advanced Science & Technology Institute (CoARE-ASTI) of the Philippine government ensures that we can move to their cloud servers (these are government services and are continuously kept running) if funding from the other sources are cut. Also, since Rice Galaxy is federated, there are tools and workflows that will continue to be hosted in collaborating Galaxy servers such as SouthGreen Bioinformatics Galaxy server in France. Lastly the tools and allied program (Open-source) are deposited in the Rice Galaxy and soon in the Galaxy Public toolshed, which allows institutions and end-users to have their instance of Rice Galaxy, complete with the tools and workflows. The main Galaxy toolshed should be stable for a relatively long time.

2nd sub-question - cost of Rice Galaxy: The system as deployed in Amazon Web Services with the current specs (t2.large, 2 x 100gb mounted volumes), using AWS calculator is ~$110 USD per month, and we get an actual billing a bit lower to this cost (~97$ US per month). The servers running at CoARE ASTI is freely hosted.

3rd & 4th subquestions , which are related: cost of data transfer of all rice VCFs from AWS to Rice Galaxy: Currently we don't support transfer of all VCFs to Rice Galaxy server, as the reviewer may know already, this is cost-prohibitive, on the Galaxy server side. We only allow the file size limits set by Galaxy for registered users (6 Gb) hence it's for analysis of a small subset of rice varieties (one or two). Copying the VCF of choice by the user into Rice Galaxy server via AWS CLI is free to the registered end-user, costs are passed on to us, added to the AWS server cost.
The reviewer's concern on the costing for server operation is a valid one, since there's a need to keep this resource available. So the model of sustainability we follow is that for end-to-end analyses (from VCF to the analyses results), we support low throughput only (fewer samples, 6GB storage limit) to accommodate the many registered users. However, we do make the tools available in the Rice Galaxy toolshed so that high throughput users can install the tools / workflows into their own computing systems. Downloading data from 3KRG S3 bucket to a user-installed system is free (only the end-users Internet connectivity cost, no additional one, is incurred), since AWS is freely hosting the 3KRG dataset in their AWS Public bucket.

3. The platform wrapped a lot of tools/apps with the Galaxy platform. Can individual tools be dockerized and/or made available via bioconda? If so, these tools can be integrated into other publicly accessible platforms like CyVerse DE and SciApps.

RESPONSE
Thank you for pointing these out. Most of the tools from the SNiPlay workflows (General, GWAS, haplotype) are already in the central toolshed and some of the corresponding softwares/executables are declared and deposited in Bioconda (plink, sNMF, tassel,...).
These workflows are also being dockerized in a dedicated Docker machine. (available here: https://quay.io/repository/valentinmarcon/docker-galaxy-sniplay).
For other tools, yes, we are in process of dockerizing other tools and will make this available, this was not included in the scope of the submitted paper but is in the scope of the Rice Galaxy working group, and we will make the links available as soon as we finish them.
We are not familiar with integration with CyVerse Discovery Environment and SciApps, but conceptually, docker container of the tools we built can be integrated with environments that allow integration of container apps, so CyVerse and SciApps might be able to integrate the containers.

4. How big is the total data source that users need to access from the Rice Galaxy?

RESPONSE
Respectfully, we don't fully understand the question but will try to respond as best as we understand it. Rice Galaxy has built-in datasets for the many rice genomes and the annotations used in the system, so this is available to users in an analysis session. External datasource is the 3K RG in Amazon S3 Public, which in total is more than 170 terabytes, but as mentioned in Q2 response, we don't allow full download of this big dataset into a Rice Galaxy session (and the size is the pre-set limit of a registered Galaxy user). So in a typical analyses session with the 3KRG, a couple of VCFs (~6gb) could be the total data downloaded from the 3KRG datasource.

Other suggestions are:

1. Is there any tutorials and working examples on using the platform with public trait data?
RESPONSE
Great suggestion, thank you. We are now using Galaxy mechanisms to have tutorials. On the home page interactive tours, we have the get 3K data video tutorials, the tutorial for SNP lifover is available in , which is where we will put most of the Rice Galaxy - specific tutorials for relevant workflows. We are committed to add more tutorials about the workflows and tools continuously.

2. Page 2 line 31, extra white space after 'phenotypic'
RESPONSE
CORRECTED, thanks!

3. Page 7, more details on how SNP lift-over works. What's the size of the flanking sequences used? How often will this workflow find the lift-over SNP or not?

RESPONSE
Thanks for reminding us of the documentation. We have updated the Rice Galaxy by sharing the tutorial page in Shared Data -> Pages section. Detailed instructions on how to optimize the lift-over workflow has been written up on the tutorial page, it's viewable here… http://13.250.174.27:8080/u/mau/p/snp-liftover-tutorial.
By default, we use 60 base flanking sequences of the SNP, to lift-over (60 bases to left of SNP, including the SNP, and 60 bases to the right, excluding the SNP) , which for most of the time, works for Nipponbare to other genomes lift-over.

4. Page 8 line 171, 'in within a JBrowse' should be changed to 'using' or 'in'
RESPONSE
CORRECTED!

5. Page 16 Line 359, 'pane' should be 'panel'
RESPONSE
CORRECTED
Thank you for the editorial corrections, we missed these, and we have addressed them accordingly.

Close
