# Supplementary material for: Rice Galaxy: an open resource for plant science
Source: Gigascience. 2019 May 18;8(5):giz028. doi: 10.1093/gigascience/giz028 (PMC6527052; doi:10.1093/gigascience/giz028)
Supplement: Response_to_Reviewer_Comments_Revision_1.pdf [file giz028_response_to_reviewer_comments_revision_1.pdf]

Author's Response To Reviewer Comments

Close

RESPONSE TO 2ND ROUND REVIEWER COMMENTS

Reviewer #2: With the clarification, my understanding is that the rice galaxy platform can at most support pulling 2 VCFs from the rice3K data hosted at Amazon, which makes it impossible to run, for example, a GWAS with a subset of the genomes. I understand that user could install the whole system locally and run their analysis. So this will be useful if everything is ready to be deployed locally, which, however, seems to be still an ongoing effort.Therefore, I would suggest to modify the manuscript with less emphasize on the demo site but more on how user can deploy the system locally. It will also be critical to clearly discuss the limitation of the demo site and which tool/workflow can be used on the demo site for what kind of analysis.

RESPONSE:
Thank you very much for highlighting these points, which are very important.
We'd like to clarify once more the matter of downloading VCFs from AWS 3KRG into Rice Galaxy. The use case this commonly addresses is for allele mining of a prior identified gene or genome region for a selected accession of interest by a rice researcher (how much variation is detected in this gene for the accession , vs the reference accession, Nipponbare). We acknowledge this might not be emphasized in the current version, thus we revised the text in the MS to clarify this point.

For GWAS analyses, we recommend a different approach, by using the pre-extracted SNP set (GWAS SNP set) across the 3K RG accessions (which is pre-computed based on LD, heterozygosity, MAF, etc. We installed tools that allow subsetting the GWAS SNP set for a selected set of accessions, and the dataset fits the resources of the Rice Galaxy server.

So as you suggested, we modified the text in the MS accordingly to address your comments, such as:

0- We distinguish between the entire Rice Galaxy system (and where to download the code to deploy), Rice Galaxy server (the deployed public reference server with rice-specific shared data), and the Rice Galaxy Toolshed (repository of Rice Galaxy tools).

1 - downloading multiple full VCFs from 3KRG is discouraged in the Rice Galaxy server. We recommend subset VCF download when using Rice Galaxy server. We mention the use case for using the VCF (full or subset) of a few 3K accessions for a gene / genome region of interest.

2- We emphasize that: for users who wish to do GWAS with 3K RG in the Rice Galaxy server, instead of multiple full VCFs, we recommend using the 1M subset SNPset in the shared data library , and instructions are written up . The 1M SNPset is derived from VCFs of the 3K RG, as described in our previous paper on SNP-Seek. The server can then handle this analyses.
For GWAS with data other than the 3K RG SNPs, we recommend uploading genotyping data in matrix format (such as hapmap).

3- We mention for each tool section whether this can be done at production scale in Rice Galaxy server itself , or best done in local/private Galaxy deployment.

4. We included a section describing the deployment Rice Galaxy server in local servers .

We hope that the current revision addresses these concerns.

Close
