# Supplementary material for: Rice Galaxy: an open resource for plant science
Source: Gigascience. 2019 May 18;8(5):giz028. doi: 10.1093/gigascience/giz028 (PMC6527052; doi:10.1093/gigascience/giz028)
Supplement: Reviewer_1_Report_Original_Submission -- Hiroaki Sakai — 8/2/2018 Reviewed [file giz028_reviewer_1_report_original_submission.pdf]

Reviewer Report

Title: Rice Galaxy: an open resource for plant science

Version: Original Submission **Date:** 8/2/2018

Reviewer name: Hiroaki Sakai

Reviewer Comments to Author:

This manuscript describes the new open access data analysis platform, Rice Galaxy, based on Galaxy framework. It is of great importance to provide the service like this to make full use of the genotyping and phenotyping data derived from the 3k rice genomes project. I had no comments on the manuscript, but several issues related to the content of the Rice Galaxy. Please note that the following comments are based on the trial from the URL http://13.250.174.27:8080/, not http://galaxy.irri.org/.
Major issues
1. Since users are not necessarily familiar with the variety IDs such as 'B001' and 'IRIS_313-10000', variety names such as 'Heibiao' and 'SUWEON 311' should be acceptable. It would be very user-friendly if Rice Galaxy converts any variety names into corresponding unique IDs.
2. It seems that only MSU gene IDs are valid to specify gene locus. If so, RAP gene IDs should also be acceptable.
3. Is there any reason for why only SOLiD data is subject to QC and manipulation tools? If Rice Galaxy outputs fastq files and/or enables users to analyze fastq files, additional tools such as QC (e.g. FASTQC), mapping to reference, and variant calling should be implemented. Otherwise, it would be better to omit the 'NGS: QC and manipulation' tools.
Minor issues
1. When users select 'Yes' for 'Filter SNP based on subpopulation' item in the 'RAVE' tool, more than one subpopulations should be able to be selected.
2. The usage of the tool 'FROM 3K RICE PROJECT - Get Subset of 3K' tells to use 'convert formats-convert BCF to VCF tool' for further analyses, but such tool is not available in the toolbox. Is 'BCFtools-bcftools view VCF/BCF' the one?
3. The tool 'Oghma' was not found in the toolbox. Is 'Genomic Prediction' the one?

Level of Interest

Please indicate how interesting you found the manuscript: Choose an item.

Quality of Written English

Please indicate the quality of language in the manuscript: Choose an item.

Declaration of Competing Interests

Please complete a declaration of competing interests, considering the following questions:

- Have you in the past five years received reimbursements, fees, funding, or salary from an organisation that may in any way gain or lose financially from the publication of this manuscript, either now or in the future?
- Do you hold any stocks or shares in an organisation that may in any way gain or lose financially from the publication of this manuscript, either now or in the future?
- Do you hold or are you currently applying for any patents relating to the content of the manuscript?
- Have you received reimbursements, fees, funding, or salary from an organization that holds or has applied for patents relating to the content of the manuscript?
- Do you have any other financial competing interests?
- Do you have any non-financial competing interests in relation to this paper?

If you can answer no to all of the above, write 'I declare that I have no competing interests' below. If your reply is yes to any, please give details below.

I declare that I have no competing interests.

I agree to the open peer review policy of the journal. I understand that my name will be included on my report to the authors and, if the manuscript is accepted for publication, my named report including any attachments I upload will be posted on the website along with the authors' responses. I agree for my report to be made available under an Open Access Creative Commons CC-BY license (http://creativecommons.org/licenses/by/4.0/). I understand that any comments which I do not wish to be included in my named report can be included as confidential comments to the editors, which will not be published.

I agree to the open peer review policy of the journal

To further support our reviewers, we have joined with Publons, where you can gain additional credit to further highlight your hard work (see: https://publons.com/journal/530/gigascience). On publication of this paper, your review will be automatically added to Publons, you can then choose whether or not to claim your Publons credit. I understand this statement.

Yes Choose an item.
