# Supplementary material for: Rice Galaxy: an open resource for plant science
Source: Gigascience. 2019 May 18;8(5):giz028. doi: 10.1093/gigascience/giz028 (PMC6527052; doi:10.1093/gigascience/giz028)
Supplement: Reviewer_2_Report_Original_Submission -- Liya Wang — 8/8/2018 Reviewed [file giz028_reviewer_2_report_original_submission.pdf]

Reviewer Report

Title: Rice Galaxy: an open resource for plant science

Version: Original Submission **Date:** 8/8/2018

Reviewer name: Liya Wang

Reviewer Comments to Author:

This paper extends the SNiPlay workflows and provides a AWS powered Galaxy platform for the Rice community to leverage the Rice variation data. This could be useful but there are several significant concerns regarding the design of the system.

1. One challenge listed in the paper is that large CPU and RAMs are needed for dealing with fairly large data matrix. However, the entire Rice Galaxy platform is powered by an AWS machine that has only 2 CPUs and 4G RAM. Is this enough for large scale computation or the system is just built for small scale computation?
2. A related question is how long will this system last. Whats the cost of the system? If user wants to download all Rice VCFs from AWS using the Rice Galaxy, what's the cost of the data transfer? Its mentioned that S3 CLI is used to copy the entire gzipped VCF file to Rice Galaxy and then get subsetted with BCFtools. Is the copy free? It will be good to know how this is handled, otherwise, the system won't last long for serving the Rice community.
3. The platform wrapped a lot of tools/apps with the Galaxy platform. Can individual tools be dockerized and/or made available via bioconda? If so, these tools can be integrated into other publicly accessible platforms like CyVerse DE and SciApps.
4. How big is the total data source that users need to access from the Rice Galaxy?

Other suggestions are:

1. Is there any tutorials and working examples on using the platform with public trait data?
2. Page 2 line 31, extra white space after 'phenotypic'
3. Page 7, more details on how SNP lift-over works. What's the size of the flanking sequences used? How often will this workflow find the lift-over SNP or not?
4. Page 8 line 171, 'in within a JBrowse' should be changed to 'using' or 'in'
5. Page 16 Line 359, 'pane' should be 'panel'

Level of Interest

Please indicate how interesting you found the manuscript: Choose an item.

Quality of Written English

Please indicate the quality of language in the manuscript: Choose an item.

Declaration of Competing Interests

Please complete a declaration of competing interests, considering the following questions:

- Have you in the past five years received reimbursements, fees, funding, or salary from an organisation that may in any way gain or lose financially from the publication of this manuscript, either now or in the future?
- Do you hold any stocks or shares in an organisation that may in any way gain or lose financially from the publication of this manuscript, either now or in the future?
- Do you hold or are you currently applying for any patents relating to the content of the manuscript?
- Have you received reimbursements, fees, funding, or salary from an organization that holds or has applied for patents relating to the content of the manuscript?
- Do you have any other financial competing interests?
- Do you have any non-financial competing interests in relation to this paper?

If you can answer no to all of the above, write 'I declare that I have no competing interests' below. If your reply is yes to any, please give details below.

I declare that I have no competing interests

I agree to the open peer review policy of the journal. I understand that my name will be included on my report to the authors and, if the manuscript is accepted for publication, my named report including any attachments I upload will be posted on the website along with the authors' responses. I agree for my report to be made available under an Open Access Creative Commons CC-BY license (http://creativecommons.org/licenses/by/4.0/). I understand that any comments which I do not wish to be included in my named report can be included as confidential comments to the editors, which will not be published.

I agree to the open peer review policy of the journal

To further support our reviewers, we have joined with Publons, where you can gain additional credit to further highlight your hard work (see: https://publons.com/journal/530/gigascience). On publication of this paper, your review will be automatically added to Publons, you can then choose whether or not to claim your Publons credit. I understand this statement.

Yes Choose an item.
